# Supplementary material for: The interactome of intact mitochondria by cross-linking mass spectrometry provides evidence for coexisting respiratory supercomplexes
Source: Mol Cell Proteomics. 2017 Dec 8;17(2):216–32. doi: 10.1074/mcp.RA117.000470 (PMC5795388; doi:10.1074/mcp.RA117.000470)
Supplement: Supplemental Data [file supp_RA117.000470_133922_0_supp_23978_fzffwf.zip › spectra_annotation/mito_spectra_annotation/103-1-5-1-11-1.pdf]

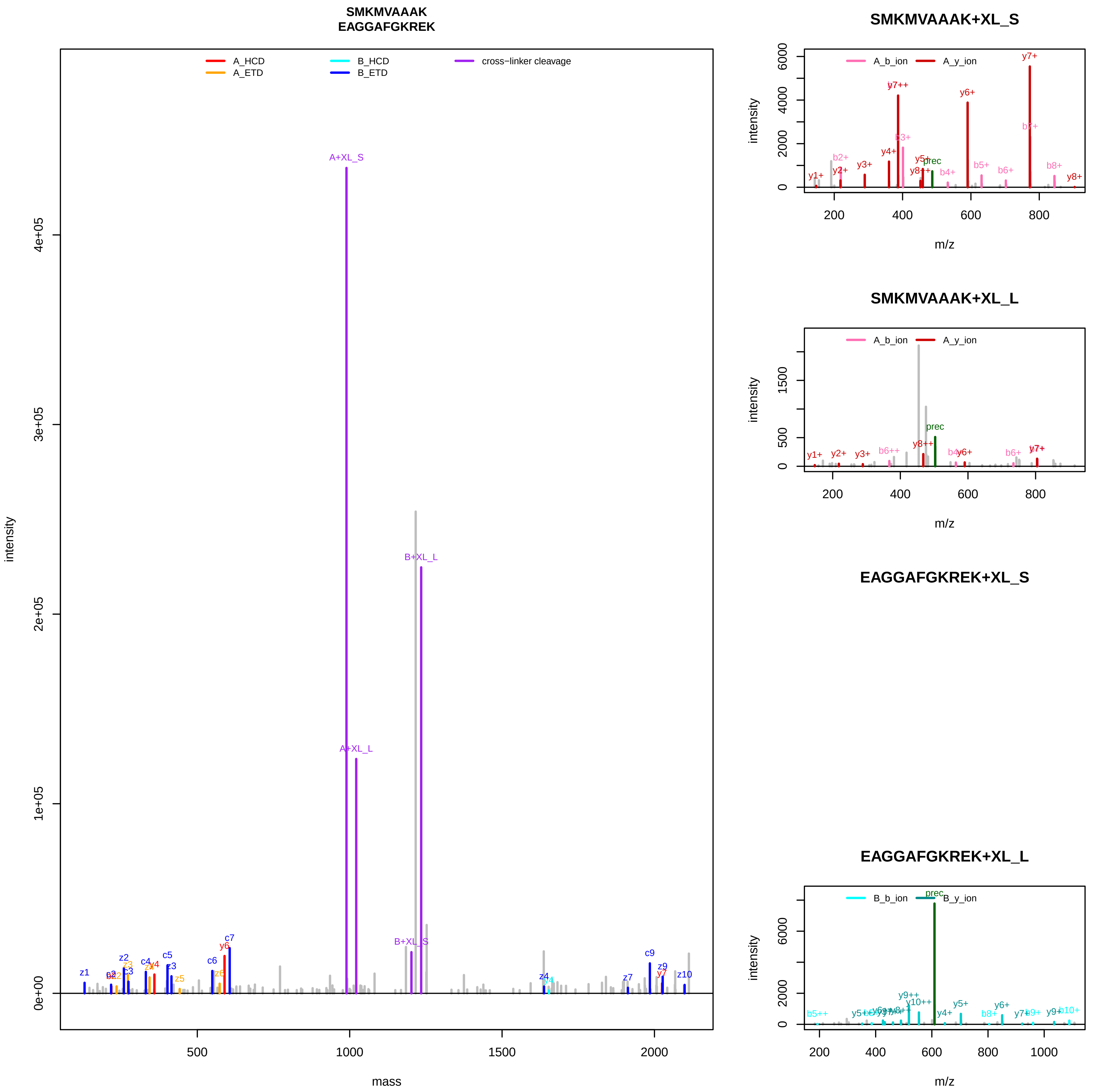

SMKMVAAAK
EAGGAFGKREK
A_HCD
A_ETD
B_HCD
B_ETD
cross−linker cleavage
A+XL_S
B+XL_L
A+XL_L
B+XL_S
intensity
mass
0e+00
1e+05
2e+05
3e+05
4e+05
500
1000
1500
2000
SMKMVAAAK+XL_S
A_b_ion
A_y_ion
y7+
y7++
y6+
b7+
b3+
y4+
y3+
b2+
y2+
y1+
y5+
prec
y8++
b4+
b5+
b6+
b8+
y8+
m/z
SMKMVAAAK+XL_L
y1+
y2+
y3+
b6++
y8++
prec
b4+
y6+
b6+
y7+
EAGGAFGKREK+XL_S
EAGGAFGKREK+XL_L
B_b_ion
B_y_ion
prec
y9++
y10++
y5+
y4+
y6+
b5++
b8+
y7+
b9+
y9+
b10+
